# Tumor Angiogenesis 2012

**DOI:** 10.1155/2012/857383

**Published:** 2012-08-02

**Authors:** Arkadiusz Z. Dudek, Kalpna Gupta, Sundaram Ramakrishnan, Debabrata Mukhopadhyay

**Affiliations:** ^1^Division of Hematology, Oncology and Transplantation, Department of Medicine, University of Minnesota, Minneapolis, MN 55455, USA; ^2^Department of Pharmacology, University of Minnesota, Minneapolis, MN 55455, USA; ^3^Department of Biochemistry and Molecular Biology, Tumor Angiogenesis, Vascular Biology and Nanotechnology Laboratory, Mayo Clinic Foundation, Rochester, MN 55905, USA

It has been more than 40 years since the initial prospect emerged that tumor angiogenesis could be targeted for cancer therapy. This proposal was based on the very simple observation that tumors are capable of stimulating new vascularization. Later the concept of an angiogenic switch was proposed; where cancer cells evolve from a dormant state into angiogenic phenotype attracting blood vessels growth and invading neighboring and distant sites. Since Folkman's landmark observations [1] a more complex picture of angiogenesis has emerged replete with a complex tumor microenvironment and interactions with multiple cell types within the tumor. Numerous anti angiogenic agents were developed successfully for the treatment of renal cell carcinoma, lung cancer, breast cancer, brain tumors, and colorectal carcinoma, but resistance to these therapies soon emerged. This stimulated additional research that led to the better understanding of the very complex interaction of tumor angiogenesis and the tumor microenvironment responsible for the lack of lasting response to angiogenesis targeted therapy.

In this issue of Tumor Angiogenesis, several review papers discuss the current status of our understanding of and therapy for ovarian carcinoma, head, and neck cancer, melanoma, glioma, and leukemia (papers by A. Amini et al., C. Carla et al., U. Adamcic et al., K. Shirai et al., S. R. Choudhury et al., and A. Trujillo et al.) 

The role of pericytes by E. Fakhrejahani and M. Toi T. Duong et al.'s contribution on the role of lymphangiogenesis enhance the spectrum of different cellular and structural components contributing to cancer progression and metastases. 

The most novel contribution emerges from the role of external stimuli in the promotion of angiogenesis. Diet as a modifier of tumor angiogenesis by W. W. Li et al. and I. Urits et al., substantiates the role of external control of microenvironment by diet, which is complemented by the observations of K. Luk et al. on pericyte-endothelial interaction influenced by morphine generally used to treat severe pain in patients with cancer. 

Insights into newer signaling pathways such as, PTEN (S. Rodriguez, and U. Huynh-Do), c-Kit (K. Rupertus et al.) and transcription factor FoxC2 (T. Kume) provide an understanding of yet to be conquered targets to efficiently treat cancer. Additionally, imaging techniques for tumor angiogenesis discussed by K. Tanaka et al. also offer novel approaches to understand tumor angiogenesis. 

The interaction between tumor vasculature and the microenvironment is reviewed by E. Fakhrejahani, M. Toi, K. Rupertus et al., and S. Niland et al., and inflammation and cancer is examined by J. I. Arias et al.

These publications provide an up to date comprehensive state of research on tumor angiogenesis. We therefore hope that this issue will help the readers understand the underlying complex biology and challenges in developing successful antiangiogenic therapy. 

*Arkadiusz Z. Dudek* *Arkadiusz Z. Dudek* *Kalpna Gupta* *Kalpna Gupta* *Sundaram Ramakrishnan* *Sundaram Ramakrishnan* *Debabrata Mukhopadhyay* *Debabrata Mukhopadhyay*
